# The role of telomere and telomerase in cancer and novel therapeutic target: narrative review

**DOI:** 10.3389/fonc.2025.1542930

**Published:** 2025-02-14

**Authors:** Temesgen Baylie, Mohammed Jemal, Gelagay Baye, Mamaru Getinet, Gashaw Azanaw Amare, Adane Adugna, Desalegn Abebaw, Zigale Hibstu, Bantayehu Addis Tegegne, Endalkachew Gugsa, Tadegew Adane, Gedefaw Getie, Baye Ashenef, Deresse Sinamaw

**Affiliations:** ^1^ Department of Biomedical Sciences, School of Medicine, Debre Markos University, Debre Markos, Ethiopia; ^2^ Medical Laboratory Science, College of Health Sciences, Debre Markos University, Debre Markos, Ethiopia; ^3^ Department of Pharmacy, College of Medicine and Health Sciences, Debre Markos University, Debre Markos, Ethiopia; ^4^ Department of Biochemistry, School of Medicine, College of Medicine and Health Sciences, University of Gondar, Gondar, Ethiopia

**Keywords:** telomere, telomere shortening, telomerase, cancer, novel therapeutic target

## Abstract

Telomeres are dynamic complexes at the ends of chromosomes that are made up of protective proteins and tandem repeating DNA sequences. In the large majority of cancer cells, telomere length is maintained by telomerase, an enzyme that elongates telomeres. Telomerase activation is seen in the majority of cancer, which permits uncontrol cell proliferation. About 90% of human malignancies show telomere dysfunction and telomerase reactivation; as a result, telomerase activation plays a special role as a practically universal stage on the way to malignancy. This review understands the structural and functional of telomere and telomerase, mechanisms of telomerase activation in oncogenesis, biomarkers and therapeutic targets. Therapeutic strategies targeting telomerase, including antisense oligonucleotides, G-quadruplex stabilizers, immunotherapy, small-molecule inhibitors, gene therapy, Telomerase-Responsive Drug Release System, have shown promise in preclinical and clinical settings. Advances in telomere biology not only illuminate the complex interplay between telomeres, telomerase, and cancer progression but also open avenues for innovative, targeted cancer therapies.

## Introduction

Telomeres are dynamic complexes at the ends of chromosomes that are made up of protective proteins and tandem repeating DNA sequences (1). Telomeres primary job is to prevent chromosome ends from being identified as double-strand DNA damage by compaction of telomere chromatin, shelterin recruitment, and structural modifications (2). When a critical telomere length is reached, shelterin will lose its binding site and telomeric DNA cannot form a protective secondary structure (3). Oncogenic cells avoid senescence and divide infinitely until numerous severely shortened telomeres trigger a crisis (4). But occasionally, rare cells manage to avoid crises and continue to proliferate by maintaining stable, albeit typically reduced, telomere lengths. Eventually, these cells develop into malignant phenotypes. The proliferative immortality of cancer cells is attained through the activation or upregulation of the normally silent human telomerase reverse transcriptase (TERT) gene (hTERT), which encodes telomerase. Rarely, telomere attrition is reversed by an additional DNA recombination mechanism known as alternative lengthening of telomeres (ALT), which avoids senescence (5). Approximately 90% of human malignancies have considerable expression of hTERT, despite the fact that it is normally repressed in almost all somatic cells. Although the exact mechanisms behind hTERT activation are still unknown, they primarily involve mutations in the hTERT promoter, modifications in hTERT pre-mRNA alternative splicing, hTERT amplification, epigenetic modifications, and/or disruption of the telomere position effect (TPE) machinery. Two cancer-specific hTERT promoter mutations, primarily C T transitions, have been linked in recent findings to the activation of telomerase in cancer cells (6). Currently, many anti-telomerase therapeutics are being evaluated in clinical trials against a variety of cancer types. The following sections will cover recent developments in the area of Structure and Function of Telomere- Telomerase, Carcinogenic mechanisms dependent on telomeres and telomerase and for the development of cancer therapies.

## Structure and function of telomere-telomerase

The protective end caps of chromosomes known as telomeres are crucial to the maintenance of our genome. Telomeres are made up of millions of repeating DNA base pairs that do not express any known proteins (7). The sequence 5′-(TTAGGG) n-3′ represents the final telomeric repeat in humans and a number of other species. The distal portion of the telomere terminates in a single-stranded G-rich segment with a shorter 5′-chain than a double strand of DNA (8). Telomeres are essential for many biological functions because they shield chromosomes against chromosomal instability and end-to-end fusions. The protective protein complex known as Shelterin binds the repeated TTAGGG sequences that make up telomeric DNA. This complex forms the telomere structure, safeguarding chromosomal ends, together with proteins involved in chromatin remodeling (7). The chromosomal ends creation of DNA loops (T-loops) and the transcription of telomeres to produce G-rich RNA. The G-rich strands 3′ end projects as the G-overhang, a single stranded overhang in the T-loop structure. This G-strand overhang invades the 5′ double-stranded telomeric duplex, looping back to generate a T-loop and the so-called D-loop. This structure ensures that loose DNA ends are housed internally within the nucleoprotein structure (9). The formation of such looped structures is an important mechanism that protects telomeres from premature degradation. Telomeres are capable of being actively transcribed while being heterochromatic, which leads to the synthesis of lengthy non-coding RNAs known as TERRAs (telomeric repeat-containing RNA). TERRA molecules are essential for controlling telomerase activity and forming heterochromatin at chromosomal ends (10).

**Figure 1:**
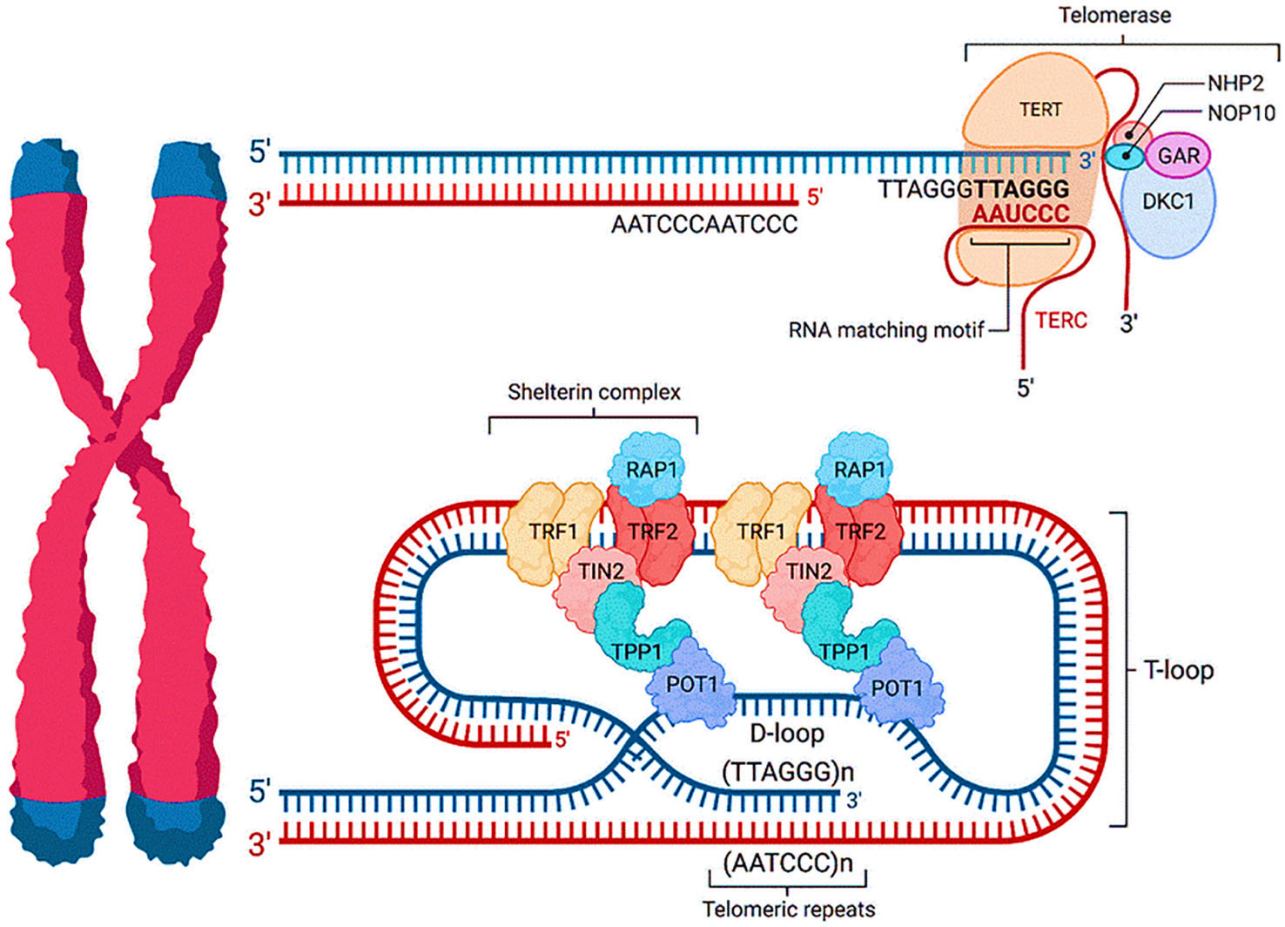
Telomere structure, shelterin complex and telomerase.

Shelterin subunits (**Figure 1**), TRF1 and TRF2, which directly recognize and bind duplex TTAGGG repeats, and POT1, which recognizes and binds single-stranded TTAGGG overhangs, make form the protein complex known as the shelterin complex, which is linked to telomeres (11). Three other shelterin proteins, TIN2, TPP1, and RAP1, link these three proteins together to produce a complex that allows DDR surveillance equipment to discriminate between genomic DNA damage sites and telomere DNA. Telomere stability is maintained by the crucial and unique functions carried out by the shelterin complex (12). For T-loop formation and ATM-mediated DDR suppression and repression from non-homologous end joining to continue, TRF2 is necessary. POT1 binds the single-stranded 3′ overhang and represses ATR-mediated DDR by blocking the recruitment of replication protein A (RPA), whereas TRF1 plays a crucial role in regulating telomeric DNA replication (13). Since TIN2 stabilizes TRF1 and TRF2 interactions with telomeric DNA and connects the TPP1/POT1 heterodimer to TRF1 and TRF2, it is critical to the overall stability of the shelterin complex. TRF2 and RAP1 interact to enhance TRF2 ability to bind to telomeric DNA selectively (14).

Long arrays of tandem TTAGGG repeats, which can range in length from 15 kb to more, make up telomeres. Two components of the shelterin complex, TRF1 and TRF2, bind to the TTAGGG/CCCTAA duplexes. In addition to bridging TRF1 and TRF2, the subunit TIN2 (TRF1-interacting nuclear factor 2) interacts with a third partner, the subunit. TPP1 interacts with POT1 (protection of telomere 1), which strengthens the cohesiveness of the nucleoprotein complex by binding the 3′ single-stranded overhang with its two OB fold domains. Lastly, the final member of Shelterin, RAP1, only interacts to TRF2. A telomerase reverse transcriptase (hTERT), an RNA component (hTERC or hTR), and a number of other protein factors, including dyskerin (DKC1), NHP2, NOP10, and GAR1, which are also components of the H/ACA ribonucleoprotein complex, make up the holoenzyme of telomerase, a retro-transcriptase.

Telomerase is a reverse transcriptase enzyme capable of extending severely short (“uncapped”) telomeres to stabilize their length. It is highly active during the early stages of human development but becomes inactivated in most adult cells. Exceptions include immune cells, germ line cells, and embryonic stem cells, where telomerase activity is maintained. Notably, telomerase is reactivated in the majority of cancers, contributing to their uncontrolled growth (15). A catalytic subunit with reverse transcriptase activity called TERT-telomerase reverse transcriptase, an RNA template called TERT-telomerase RNA component that has a sequence complementary to the telomere’s sequence, and accessory proteins like discerin, NHP2 ribonucleoprotein, NOP10 ribonucleoprotein, and GAR1 (localization factor) make up the enzyme telomerase (16). The protein complex and TERC are bound by the TERT subunit. Telomerase interacts with the telomere-localizing protein TPP1 to attract it to single-stranded telomeric DNA. The ATPases pontin and reptin, the chaperones HSP90 and p23, a WD-repeat-containing protein 79 known as TCAB1, and other components are also involved in this process. The telomerase-telomere complex is stabilized by the TERC-blinding protein, SRSF11 (17). The transcription factors c-MYC and SP1 must bind to the E-box (5′-CACGTG-3′) and five GC boxes (5′-GGGCGG-3′) in order to induce the production of TERT mRNA (18).

The transcriptional reactivation of TERT in cancer cells is mediated by many signaling pathways, primarily c-MYC, NF-κB, and B-Catenin. Furthermore, through phosphorylation, the PI3K/AKT kinase pathway increases TERT activity at the posttranslational level. When c-MYC binds to the E-box (5′-CACGTG-3′) in the TERT promoter region, TERT is activated. The activation of TERT transcriptional activity, stabilization of c-MYC levels on chromatin, c-MYC ubiquitination, and proteosomal degradation lead to an increase in vascular cell survival and stimulation of c-MYC-dependent oncogenesis potential (19). Through NF-κB binding sites in the TERT promoter that are specific for p50 and p65, NF-κB regulates the transcription of TERT.In addition to causing TERT transcription activities, this pathway also modifies TERT nuclear translocation, recruits IL6 and TNF alpha, represses ROS-dependent activation, and increases MMP. These actions lead to the suppression of inflammation, ROS-dependent activation, and the advancement of cancer. TERT regulation also involves the Wnt/B-Catenin pathway (20).

## Telomerase: the key telomere length maintenance mechanism

Telomerase maintains telomere length (TL) through a series of molecular events. These include the transport of (human telomerase reverse transcriptase) hTERT protein into the nucleus, the assembly of (human telomerase RNA) hTR and hTERT with accessory components within the nucleus, and the recruitment of telomerase to telomeres during DNA replication. The hTERT is a catalytic subunit, while hTR serves as a template for telomere elongation (21). Other proteins, dyskerin, NHP2, NOP10, and GAR1 (localization factor), that are linked to the H/ACA family of short nucleolar RNAs, are also part of the telomerase holoenzyme and are required for the pseudo uridylation that occurs during the post-transcriptional modification of RNAs. The CAB-box sequence in the hTR is bound by WD-repeat-containing protein 79, or TCAB1, that then directs the telomerase holoenzyme to the Cajal bodies attached to the nucleolus (22). These molecular players are chaperones HSP90 and p23, as well as the ATPases pontin and Reptin, known to bind to telomerase’s major two subunits. This allows for the proper assembly and stabilization of telomerase into its holoenzyme form (23); the most biologically plausible human telomerase model would involve a process that could yield the scaffold assembly platform for dyskerin, the nascent forms of hTR transcripts, together with pontin and Reptin. Subsequently, the telomerase RNP particle then interacts with NHP2 ribonucleoprotein, NOP10 ribonucleoprotein, a nuclear assembly factor ribonucleoprotein (NAF1), and the dyskerin H/ACA motif-binding complex. Subsequently, NAF1 is removed by hTR and GAR1 added, forming a physiologically stable hTR-H/ACA-RNP complex. The catalytically active telomerase RNP is produced when hTERT binds to two structurally distinct hTR domains (CR4/CR5) after the hTR 3′-hairpin CAB-box sequence recruits TCAB1 (24).

**Figure 2:**
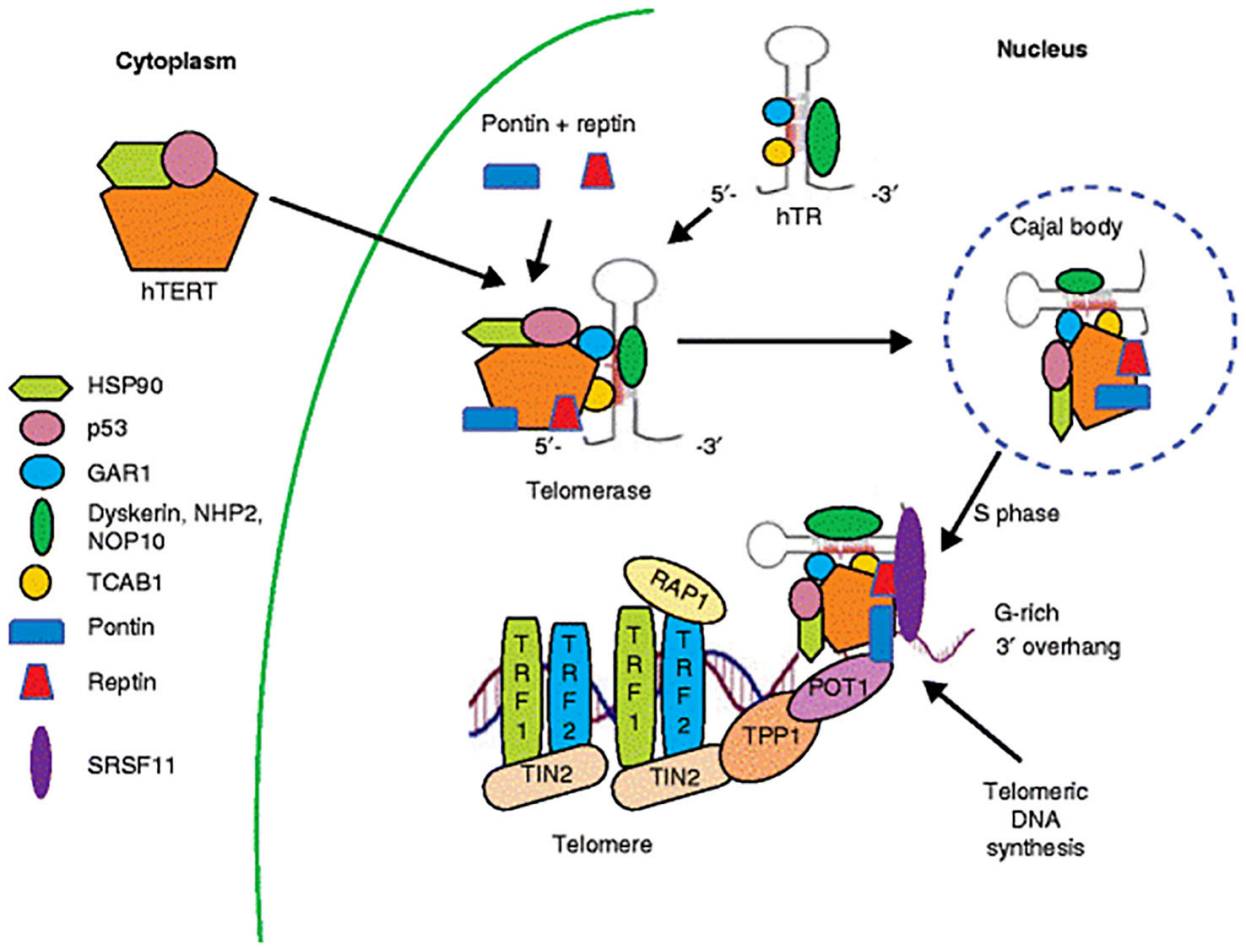
The main mechanism for maintaining telomere length.

Telomerase can only be recruited to telomeres after the replication fork remodels the protected DNA 3′ ends during the S phase of the cell cycle (**Figure 2**). This process is dependent on protein-protein interactions involving the DAT (dissociates the activities of telomerase) domain of hTERT. These interactions distinguish its activity from an *in vitro* setting and allow it to interact with shelterin complex components, specifically TPP1 and POT1, which are essential for telomerase recruitment. This part of TPP1 is an amino acid patch known as the Tel patch located in the N-terminal oligonucleotide/oligosaccharide-binding (OB)-fold domain that directly interacts with the telomerase DAT domain (25). It also contains a C-terminal domain that binds to TIN2 and a central domain that directly interacts with POT1. Thus, the interaction between the DAT domain of hTERT and shelterin components ensures proper telomerase placement at the 3′ end of DNA for synthesis and processivity of telomeric repeats. SRSF11 is a new TERC-binding protein that is involved in the process of loading telomerase onto the telomeres. This therefore allows for stable interaction between the enzyme and the telomere overhang, such that the DNA 3′ end is correctly positioned near the active site of the enzyme for nucleotide addition. It thus plays a critical role in the maintenance of telomere integrity, and there may be serious implications for cell aging and diseases such as cancer resulting from disruptions in this process (26).

In eukaryotic organisms, the cellular ribonucleoprotein enzyme complex known as telomerase is responsible for catalyzing the extension of telomeric DNA. A number of processes are involved in telomerase action, such as the telomerase complex’s construction, intracellular trafficking, and recruitment to telomeres. Human telomerase is made up of the auxiliary proteins dyskerin, NOP10, NHP2, and GAR1, as well as hTR (hTERC—a template functional RNA) and hTERT (the catalytic protein component with reverse transcriptase activity). In the cytoplasm, the hTERT protein binds to p23 and HSP90 before entering the nucleus. NHP2, GAR1, NOP10, and dyskerin form a complex with nascent hTR transcripts. This complex is bound to the hTERT +p23 + HSP90 complex via Reptin and Pontin. Then, TCAB1 binds to this assembling complex and directs it to the nucleus’ Cajal bodies. Through interactions between the DAT domain of hTERT and the shelterin complex components TPP1 and POT1, telomerase is recruited to telomeres during the S phase of the cell cycle. For DNA synthesis, SRSF11 maintains the telomerase enzyme complex’s attachment to the telomere overhang.

## Telomerase used as cancer biomarker

Telomerase activity is highly upregulated in most of cancers that have been surveyed across in the vast majority of malignancies, including breast, colon (27, 28), melanoma (29), oral (30), ovarian (31), pancreas (32), prostate, and soft tissue cancer (33). Because of phenotypic difference between malignant and normal/benign tissues, telomerase used as a universal cancer biomarker for prognosis and diagnosis of cancer (34). The telomeric repeat amplification protocol (TRAP), a technique for assessing telomerase activity. This assay consists of two steps: an amplification reaction where any TS oligo extended by telomerase in the lysate is PCR amplified for detection, and an extension reaction where a “telomerase substrate” (TS) oligonucleotide mimicking a 3’ telomere end is combined with cell lysate containing telomerase (35). As little as 1–10 telomerase positive cells or 0.01% positive cells in a mixed population can be found with this extremely sensitive assay. Telomerase activity has been established using TRAP in the great majority of cancer types, but not in certain normal somatic tissues. However, TRAP activity was found in germ-line tissues, and weak activity was reported in specific stem cell populations and peripheral blood leukocytes (36).

TERT mRNA expression level has been investigated as a biomarker in addition to telomerase enzymatic activity since it has been shown to be the rate-limiting factor of telomerase activity in a number of cancers, including breast, non-small cell lung, and urothelial cancer (37). The expression of TERT mRNA is directly linked to the activation of telomerase, which is essential for tumor cell proliferation and immortality. The presence of plasma TERT mRNA was detected in patients with prostate cancer, with sensitivity at 85%, specificity at 90%, positive predictive value at 83%, and negative predictive value at 92%, making it a reliable diagnostic and prognostic tool (38). TERC, the telomerase RNA, has been thought to be an unsuitable cancer marker because, unlike TERT mRNA, it is often expressed widely and occasionally even in the absence of telomerase activity. Nonetheless, oesophageal cancer, gastric carcinomas, and astrocytomas all had noticeably higher TERC RNA levels (39). Although TERC expression is not tumour-specific its highly expressed level may be indicative of the presence of tumor in some cancer types. It is not detectable in benign tissue but is reported to be in 17/18 cases by real-time qPCR in all breast cancer tumors (40). qPCR, being a high sensitivity technique for amplifying the TERC RNA copy, is more sensitive and was indeed detectable with small amounts of tumor tissue. Recently, excreted urine from bladder cancer patients was tested for telomerase activity, TERT mRNA, and TERC telomerase RNA with a range of sensitivities and specificities (41). The utilization of urine as a non-invasive sample has the potential to allow for early detection of bladder cancer. Among these three techniques compared in urine from 200 bladder patients, the sensitivity of TERT mRNA, TERC, and telomerase activity for detecting bladder cancer were 96%, 92%, and 75%, respectively (42).

## Telomerase as a therapeutic target in cancer treatment: exploring inhibition strategies

Developing effective treatments to combat cancer, telomerase is supposed to be a key target (43). Maintaining telomere lengths that are comparably long to those of cancer cells. These traits offer a benefit that guarantees a low chance of telomere shortening in healthy cells. Anti-telomerase medications primarily target cancer cells to cause apoptosis and cell death, with little to no effect on normal cells because somatic cells express very little or no telomerase (44). The majority of malignancies (85%–90%) exhibit telomerase activity. As a result, telomerase suppression presents an appealing target for cancer therapy. Numerous strategies, including antisense oligonucleotides, G-quadruplex stabilizers, immunotherapy, small-molecule inhibitors, gene therapy, Telomerase-Responsive Drug Release System have been developed in the hunt for telomerase inhibitors (45).

### Oligonucleotides

The use of antisense oligonucleotides to prevent the translation of mRNA with a sequence complementary to sense RNA was the first description of the antisense oligonucleotide technique for targeting telomerase (69). Targeting the catalytic component of telomerase (hTERT), oligonucleotides are made up of short sequences of single-stranded DNA (SS-DNA) that bind to the RNA template in a complementary manner to limit telomerase activity. GRN163L is currently the most effective oligonucleotide. The treatment of human bladder cancer cells *in vitro* with oligonucleotides targeted to hTERT inhibits the cells’ ability to proliferate. Small interfering RNA (siRNA) operates through a mechanism involving small double-stranded RNA molecules that form an RNA-induced silencing complex (RISC). This complex binds to target mRNA and cleaves it, effectively silencing gene expression (46). The technique very well for short-term hTERT knockdown because the cells break down the dsRNA. Using plasmid constructs that exogenously express short hairpin RNA sequences to the hTERT transcript, RNA interference (RNAi) of hTERT has also been effective. This method permits long-term and permanent gene knockdown and acts as an alternative to gene therapy via viral vectors (47). Long-term hTERT knockdown can also be achieved by using retroviral vectors that express short hairpin RNA specific to a section of the hTERT transcript. Telomerase activity is competitively inhibited by Imetelstat. A 13-mer N3′–P5′ thiophosphoramide oligonucleotide is included in it, and a 5′-thio-phosphate group covalently bonds it to a palmitoyl moiety. The thiophosphonate backbone of Imetelstat possesses a variety of physicochemical characteristics, including excellent water solubility, metabolic stability, resistance to nuclease activity, and stability in the formation of RNA duplexes (48, 49). The sequence 5′-palmitate- TAGGGTTAGACAA-NH2- 3′ of Imetelstat binds to a complementary 13-nucleotide region of h TR, which exhibits great specificity and affinity to the active site of telomerase enzyme (12, 50).

### Anti-telomerase immunotherapy

The main idea behind the hTERT-specific T cell therapy for anticancer immune cancers is to target telomerase positive malignancies. In actuality, the use of proteasomes to break down telomerase results in the production of telomerase protein fragments that are produced as antigens on the surface of tumor cells by the human leukocyte antigen (HLA) class-1 pathway (51). Thus, cytotoxic T lymphocytes can target antigenic telomerase epitopes to eliminate tumor cells. Telomerase-specific epitopes can activate antigen-presenting cells to assault tumor cells or trigger CD4+ or CD8+ cytotoxic T-lymphocyte responses. By sensitizing the immune system to the tumor cell’s presentation of hTERT peptides, this therapy produces increased anticancer effects by activating and generating CD8+ cells that are specific to h TERT (12). It’s interesting to note that cancer patients can stimulate anti-telomerase immune responses by administering three key vaccines: GV1001L, Vx001, and GRNVACI. It’s interesting to note that in both mice and humans, immunization with autologous dendritic cells transfected with hTERT mRNA also produces CD4+ and CD8+ T responses (52).

### TERT-targeted peptide vaccines

Out of all the TERT vaccinations, GV1001 is the most advanced. It triggers certain T cell reactions in melanoma, NSCLC, and pancreatic cancer. Strong CD4+ and CD8+ T cell responses as well as the activation of cytotoxic T lymphocytes (CTL) are produced by this MHC class II restricted peptide vaccination (53). Prostate and kidney cancer patients have tested for GX301, a vaccine made up of four peptides generated from TERT that is more effective than single-peptide vaccines. These studies’ findings suggest that multi-peptide vaccinations are more successful than single-peptide vaccines because they boost the immune response in a greater number of responders (54). There has been evidence of immune responses to the UV1 or Vx-001 vaccines in NSCLC and prostate cancer, respectively. The first of them decreased prostate-specific antigen (PSA) levels in 64% of patients with metastatic prostate cancer and elicited an immunological response in 85.7% of patients. In NSCLC, the second vaccination produced a robust TERT-specific immune response and had a favorable tolerance profile (55).

### DNA vaccines

Recombinant DNA technology can be used to generate the genome encoding the TERT peptide. These genomes can be inserted onto plasmids and transmitted to antigen-presenting cells, increasing T lymphocyte epitope presentation effectiveness. While INVAC-1 is a plasmid that codes for the inactive version of TERT, phTERT is a DNA-based vaccination that codes for TERT (16). In the HPV-related and melanoma-related tumor models, respectively, the phTERT and INVAC-1 vaccinations reduced tumor growth and extended survival (56).

### Small molecule inhibitors

By non-competitively attaching to the active site of TERT, the non-nucleotide small molecule chemical known as BIBR1532 acid specifically suppresses telomerase activity. Despite the positive outcomes of preclinical research on cell lines from breast and prostate cancer (57).

### Targeting G-quadruplex

The telomerase enzyme is prevented from accessing the telomeric 3′-overhang by the G-quadruplex. Experimental data indicate that the G-quadruplex structure exists *in vivo* and *in vitro*, where it is involved in 3′ end protection (58). The creation of compounds that stabilize G-quadruplex structure is prompted by the importance of G-quadruplex structure in telomere stabilization. 3,6,9-trisubstituted acridine is the trisubstituted acridine BRACO-19.G-quadreplex ligand -3,6-bis(3-pyrrolodinopropionamido) acridine inhibits telomerase access and causes telomere uncapping and telomeric fusion (59). RHPS4 (3,11-difluoro-6,8,13-trimethyl-8H-quino[4,3,2-kl] acridinium methosulfate) is a pentacyclic acridine that is another G4 ligand that may have anticancer properties. Treatment with RHPS4 caused telomere dysfunction, toxicity, and DNA damage signaling in melanoma cells, but not in normal fibroblasts. Teloestatin, a G-quadruplex stabilizing molecule, was isolated from Streptomyces anulatus 3533-SV4 and demonstrated both apoptosis and telomere shortening (60).

### Gene therapy

One of the most well-known gene therapy compounds is imetelstat (GRN163L), which has been previously described. A chimera gene, or the coding sequence of a proapoptotic protein regulated by the TERT gene promoter, is one of the alternative tactics. An attenuated adenovirus-5 vector called telomelysine causes cancer cells that overexpress TERT to lyse. Utilizing genetically altered virus vectors that encode a cytotoxic prodrug activating enzyme is a further strategy (61).

Telomerase inhibitors derived from natural products and microbial sources:

It has been determined that natural materials with the ability to suppress telomerase could be used as chemotherapeutic drugs to treat cancer. Polyphenols (curcumin, quercetin, resveratrol, and tannic acid), alkaloids (boldine and berberine), terpenoids (pristimerin and oleanane), and xanthones (gambogic acid and gambogenic acid) are examples of naturally occurring plant chemicals that have telomerase-inhibiting properties (62). Oleic acid is a monounsaturated fatty acid that is found in both vegetable and animal oils. It is categorized as omega-9 and has been shown to inhibit human telomerase (61). Microorganisms called actinomycetes spp. have rings of benzofuran and benzodipyrane, which have telomerase-inhibiting properties. Recently, telomerase inhibitory action of robomycins derived from Streptomyces collinus has been investigated (63).

### Targeting telomeric cap

Many studies have shown that TRF1 and TRF2 are overexpressed in a variety of malignancies. TRF1 and TRF2 prevent end-to-end fusion of chromosomes by binding to the telomeric double-stranded 5′-TTAGGG-3′ repeat (64). Because TRF1 and TRF 2 are crucial for genomic stability, it may be more necessary to target them in order to cause genomic instability in malignant cells, which ultimately results in cell death. Considering this, we have demonstrated that focusing on TRF1 and TRF2 in RCC results in both apoptosis and cell cycle arrest (65). The Maria Garcia-Beccaria group has demonstrated that focusing on TRF1 abrogation results in a significant decrease in the quantity and size of malignant lung cancer (66). POT1, stabilizes the telomeric structure by binding to the single strand overhang of the telomeric sequence with a very high degree of sequence specificity. When anti-POT1 siRNAs were used to treat breast cancer cells, the function of POT1 in telomere protection was determined. Treatment with POT1 siRNA causes telomere disruption, activation of apoptosis, and increased expression of p53 and the pro-apoptotic protein Bax (67). Telomere end fusion and chromosomal instability were caused by POT1 gene deletion in mice provides more evidence for POT1’s potential involvement in carcinogenesis. Research has demonstrated that the loss of POT 1 causes a temporary DNA damage response at chromosomal ends (68).

### Inhibition of signalling pathways

The MAP kinase-mediated signaling pathways have the potential to activate the hTERT gene. For instance, blocking of this route may be a novel strategy to lower hTERT expression and telomerase activity. EtS and AP-1 may be involved in MAP kinase signaling to the hTERT gene (69). Zheng et al. found that the Tetra-Pt derivative of cisplatin, independent of telomerase, could observably suppress tumor growth in ALT-activated U2OS cancer cells by inhibiting the ALT pathway (70).

### Downregulation of TERT expression

Shortening telomere lengths can be accomplished by downregulating TERT expression through the use of gene therapy technology. 5-aza-2′-deoxycytidine (DAC), a DNA demethylating agent, shortened telomeres by suppressing TERT expression, which was brought on by a reduction in c-myc binding to the hTERT promoter (71). The downregulation of hTERT resulted in a notable reduction in telomere length, which in turn inhibited the growth and carcinogenic potential of A549 cancer cells (72). A recombinant retrovirus vector containing the whole hTERT antisense complementary DNA was produced. It is evident that the vector system may suppress telomerase activity and hTERT gene expression, ultimately slowing the growth of the ovarian tumor and extending the mice’s survival period. The ZD55-hTERT and PEGylated carboxymethyl chitosan/calcium phosphate hybrid anionic nanoparticles containing hTERT siRNA have the ability to significantly quiet hTERT, hence impeding the growth of tumors (73).

## Active agents in clinical trials

Since 2010, as indicated in (**Table 1**), several telomere-targeted therapies have been evaluated in clinical trials for a variety of cancer types. Telomere has currently emerged as a promising target in cancer therapy. Drugs targeting telomeres that have been approved in clinical trials primarily fall into two groups: compounds used in chemotherapy and immunotherapy (74). Telomerase-specific oncolytic adenoviruses, like OBP-301 and KH901; hTERT peptide vaccines, like GV1001, UCPVax, VX-001, UV1 and GX301, hTERT peptide, and hTERT and survivin multi-peptide; hTERT DNA vaccines, like INVAC-1, V934/V935 and INO-1400/1401/5401; hTERT mRNA vaccines (75); dendritic cell vaccines, like hTERT RNA transfected DCs and hTERT peptide pulsed DCs; and transgenic lymphocyte immunization vaccines against telomerase (76). Chemotherapeutics are further subdivided between nucleoside analogue 6-thio-2’-deoxyguanosine and telomerase inhibitors like imetelstat and KML-001, which induce telomere dysfunction (43). 6-thio-dG (THIO) induces telomerase mediated telomeric DNA damages that are sensed by dendritic cells and activates the host cytosolic DNA sensing STING/interferon I pathway, resulting in tumor-specific CD8+ T cell activation. In addition, 6-thio-dG overcomes resistance to checkpoint blockade in advanced cancer mode (77, 78). Imetelstat was shown in multiple clinical trials to suppress telomerase activity in tumor cells and peripheral blood mononuclear cells (PBMCs), however it had serious adverse effects in children with recurrent central nervous system (CNS) malignancies (76). Imetelstat improved median progression-free survival (PFS) and overall survival (OS) in patients with non-small cell lung cancer (NSCLC) (79).

**Table 1:** Clinical trials of telomere-related anti-cancer therapeutics.

Intervention	Indication	Study Start Year	Phase	Main Results	Ref
GV1001 and temozolomide	Advanced malignant melanoma	2010	I/II	Improved progression-free survival (PFS) and overall survival (OS); partial responses in some patients.	(80)
GV1001 (hTERT: 611–626); p540 (hTERT:	Cutaneous melanoma	2011	I	Partial responses and stabilization in some patients.	(81)
GV1001 (after GM-CSF)	Inoperable stage III non-small cell lung cancer	2012	III	Improved disease control, with some patients showing responses.	(82)
Imetelstat sodium	Children with refractory or recurrent solid tumors or lymphoma	2011	I	Some partial responses and disease stabilization.	(83)
Imetelstat	Children with refractory or recurrent solid tumors	2013	I	Disease stabilization in a few cases, with manageable side effects.	(84)
GRN163L and Trastuzumab	HER2^+^ breast cancer	2010	I	Enhanced anti-tumor activity and improved PFS.	(85)
Imetelstat; paclitaxel with or without Bevacizumab	Locally recurrent or metastatic breast cancer	2010	II	Some patients showed disease stabilization and response.	(86)
Imetelstat	Children with recurrent or refractory central nervous system malignancies	2017	II	Modest disease control and partial responses.	(85)
KML-001 (sodium metaarsenite) and cisplatin	Advanced solid tumors	2010	I	Partial responses and disease stabilization in refractory solid tumors.	(87)
THIO (6-Thio-2’-Deoxyguanosine); Cemiplimab	Advanced non-small cell lung cancer	2022	II	Some complete responses and prolonged survival in a subset of patients.	(88)
UCPVax and atezolizumab	Human papilloma virus positive cancers	2019	II	Enhanced immune responses and tumor regression in a subset of patients	(89)
Nivolumab and ipilimumab with or without UV1	Inoperable malignant pleural mesothelioma after first-line platinum-based chemotherapy	2020	II	Improved PFS and OS in some patients.	(90)
UCPVax and nivolumab	Advanced non-small cell lung cancer	2020	II	Some responses and improved survival in advanced cases.	(91)
OBP-301 (telomerase specific replication- competent oncolytic adenovirus)	Unresectable metastatic melanoma	2016	II	Reduction in tumor size and viral replication in tumors.	(92)
UCPVax; radiotherapy and temozolomide	Glioblastoma	2020	II	Prolonged PFS and enhanced immune responses.	(93)
OBP-301 (telomelysin) with radiotherapy	Esophageal cancer	2017	I	Some tumor reduction and improvement in disease control.	(94)
Telomelysin (hTERT promoter driven modified	Solid tumors	2010	I	Tumor shrinkage in select patients, promising early results.	(95)
INO-5401; INO-9012; cemiplimab (REGN2810); radiation and chemotherapy	Newly-diagnosed glioblastoma	2018	I/II	Promising survival benefit with enhanced immune responses.	(96)
OBP-301; carboplatin; paclitaxel and radiation therapy	Locally advanced esophageal and gastroesophageal cancer	2020	I	Modest disease control and tumor response.	(97)
mRNA (MUC1, CEA, Her-2, telomerase, surviving and MAGE-A1) and GM-CSF	Stage IV renal cell cancer	2011	I/II	Some immune responses and disease stabilization	(98)
GV1001 (after radiotherapy and docetaxel)	Non–small cell lung cancer	2011	I/II	Improved survival and disease control in a subset of patients.	(99)
UCPVax (emulsified in montanide ISA-51)	Metastatic non-small cell lung cancer	2016	I/II	Enhanced immune responses and disease control.	(100)
GV1001 (after gemcitabine and capecitabine)	Advanced pancreatic ductal adenocarcinoma	2015	III	Some partial responses and disease stabilization.	(101)
atezolizumab and bevacizumab	Unresectable hepatocellular carcinoma	2022	II	Improved OS and PFS in patients with advanced HCC.	(102)
UV1; GM-CSF and ipilimumab	Unresectable or metastatic malignant melanoma	2014	I/II	Immune responses and some disease stabilization.	(103)
INO-1400 or INO-1401 (hTERT)	Solid tumors	2016	I	Immune responses and partial responses in solid tumors.	(104)
UV1 and GM-CSF	Men with metastatic hormone-naïve prostate cancer	2013	I/II	Some partial responses and immune activation.	(105)
GX301 (emulsified in montanide ISA-51 and imiquimod 5% cream)	Castration-resistant prostate cancer	2014	II	Tumor regression and disease stabilization in select patients.	(54)
INO-5401 (WT1, PSMA and hTERT); INO- 9012 (IL-12) and atezolizumab	Locally advanced unresectable or metastatic/recurrent urothelial carcinoma	2018	I/II	Enhanced immune responses and disease stabilization.	(106)
TERT and surviving multi-peptide vaccine	Myeloma	2011	I/II	Some partial responses and immune activation.	(107)
INVAC-1	Solid tumor	2020	I	Early results indicated immune responses and disease control.	(108)
hTERT mRNA DCs	Metastatic prostate cancer	2010	I/II	Immune activation and tumor regression in a subset of patients.	(109)

## Conclusions

This study concludes that understanding of telomere and telomerase structure and function, and of their mechanisms of action, holds the greatest potential for the discovery of novel cancer therapies. Because shelterin and dynamic telomeres, crucial for the survival and proliferation of cancer cells, depend on the modulation of telomerase activity entailing telomerase and shelterin protein interactions and substrate recruitment to the active site of telomerase, this may prove to be a very good strategy. The molecular mechanisms of genetic and epigenetic regulation that activate one of the hallmarks of cancer, human telomerase reverse transcriptase, play very important roles in the initiation of cancer and tumor progression. These features provide a strong foundation for developing targeted therapeutics to treat cancer by targeting specific telomere maintenance mechanisms. Several strategies have been employed in telomere-related therapeutics, such as downregulating TERT expression, reducing telomerase recruitment to telomeres, inducing telomere dysfunction, and interfering with the alternative lengthening of telomeres (ALT) pathway. Several telomere-targeting agents have been developed, including antisense oligonucleotides that block the expression of TERT, the enzyme responsible for telomerase activity; G-quadruplex stabilizers that prevent telomerase from elongating telomeres; small-molecule inhibitors that inhibit telomerase activity or prevent its recruitment to telomeres; and immunotherapies that trigger immune responses against cancer cells by inducing telomere stress. Gene therapy with telomerase promoter-driven suicide genes selectively kills cancer cells overexpressing telomerase, while ALT inhibitors block the alternative lengthening of telomeres pathway used by some cancers. These agents offer the potential for selective, less toxic treatments for cancer, often in combination with other therapies such as chemotherapy or immunotherapy. However, for optimal efficacy, several challenges must be overcome, including tumor heterogeneity, timing of treatment, and potential for side effects. Despite these challenges, telomere-targeting agents represent a promising approach in cancer therapy by directly attacking the mechanisms that allow cancer cells to avoid normal aging processes and continue to proliferate.
